# Weak population structure of the Spot‐tail shark *Carcharhinus sorrah* and the Blacktip shark *C. limbatus* along the coasts of the Arabian Peninsula, Pakistan, and South Africa

**DOI:** 10.1002/ece3.4468

**Published:** 2018-08-29

**Authors:** Dareen Almojil, Geremy Cliff, Julia L. Y. Spaet

**Affiliations:** ^1^ Department of Zoology University of Cambridge Cambridge UK; ^2^ KwaZulu‐Natal Shark Board Umhlanga, South Africa and School of Life Sciences University of KwaZulu‐Natal Durban South Africa; ^3^ Red Sea Research Center Division of Biological and Environmental Science and Engineering King Abdullah University of Science and Technology Thuwal Saudi Arabia

**Keywords:** elasmobranchs, genetic diversity, Indian Ocean, low significant *F*_ST_, microsatellites, philopatry, Tethys Sea closure

## Abstract

The increase in demand for shark meat and fins has placed shark populations worldwide under high fishing pressure. In the Arabian region, the spot‐tail shark *Carcharhinus sorrah* and the Blacktip shark *Carcharhinus limbatus* are among the most exploited species. In this study, we investigated the population genetic structure of *C. sorrah* (*n* = 327) along the coasts of the Arabian Peninsula and of *C. limbatus* (*n* = 525) along the Arabian coasts, Pakistan, and KwaZulu‐Natal, South Africa, using microsatellite markers (15 and 11 loci, respectively). Our findings support weak population structure in both species*. Carcharhinus sorrah* exhibited a fine structure, subdividing the area into three groups. The first group comprises all samples from Bahrain, the second from the UAE and Yemen, and the third from Oman. Similarly, *C. limbatus* exhibited population subdivision into three groups. The first group, comprising samples from Bahrain and Kuwait, was highly differentiated from the second and third groups, comprising samples from Oman, Pakistan, the UAE, and Yemen; and South Africa and the Saudi Arabian Red Sea, respectively. Population divisions were supported by pairwise *F*_ST_ values and discriminant analysis of principal components (DAPC), but not by STRUCTURE. We suggest that the mostly low but significant pairwise *F*_ST_ values in our study are suggestive of fine population structure, which is possibly attributable to behavioral traits such as residency in *C. sorrah* and site fidelity and philopatry in *C. limbatus*. However, for all samples obtained from the northern parts of the Gulf (Bahrain and/or Kuwait) in both species, the higher but significant pairwise *F*_ST_ values could possibly be a result of founder effects during the Tethys Sea closure. Based on DAPC and *F*_ST_ results, we suggest each population to be treated as independent management unit, as conservation concerns emerge.

## 1 INTRODUCTION

With an increasing number of conservation challenges and species under threat, population genetics offer a noninvasive tool to uncover otherwise unattainable information (Allendorf & Waples, 1996; Van Wijk et al., 2013). The identification of genetic structure is fundamental in determining the extent of reproductive isolation between populations (Hartl, 1988) and can have direct implications in designing effective protection plans.

In sharks, studies of genetic structure have shown subdivision on different geographic scales, ranging from small‐scale genetic structure across less than hundreds of kilometers (Gaida, 1997), to large‐scale genetic structure between regions separated by ocean basins (Benavides et al., 2011; Daly‐Engel et al., 2012; Duncan, Martin, Bowen, & De Couet, 2006; Portnoy, McDowell, Heist, Musick, & Graves, 2010; Schultz et al., 2008), to worldwide panmixia (Castro et al., 2007; Hoelzel, Shivji, Magnussen, & Francis, 2006). The genetic structure observed in different shark species is believed to depend on hard and soft barriers to gene flow. Hard barriers result from ancient events creating a physical landmass barrier to oceanic gene flow (e.g., the terminal Tethyan Event and the Isthmus of Panama, which separated the Indian and Atlantic Oceans and the Pacific and Atlantic Oceans, respectively). Soft barriers to gene flow are those related to a species’ biology and behavior or invisible physical factors such as water currents or temperature (Cowman & Bellwood, 2013).

In sharks, biological and behavioral factors reported to influence genetic structure are vagility and reproductive behavior. Vagility is associated with body size, and a positive correlation has been found between body size and dispersal range (Mejía‐Falla & Navia, 2011). This is supported by the finding that large species [>3 m total length (TL)] often have circumglobal distributions, for example, the whale shark *Rhincodon typus* (Castro et al., 2007) and the basking shark *Cetorhinus maximus* (Hoelzel et al., 2006). Reproductive behaviors such as female philopatry can lead to restricted connectivity in some species, for example, the white shark *Carcharodon carcharias* (Pardini et al., 2001) and the blacktip shark *Carcharhinus limbatus* (Keeney & Heist, 2006). Physical factors associated with shark genetic structure are deep water (Benavides et al., 2011; Duncan et al., 2006; Ovenden et al., 2011), warm equatorial waters (Chabot & Allen, 2009; Mendonça, Oliveira, Gadig, & Foresti, 2011; Veríssimo, McDowell, & Graves, 2011; West & Stevens, 2001), and cold water temperatures (Keeney & Heist, 2006; West & Stevens, 2001).

The Arabian region has long been recognized as a global hotspot of marine biodiversity (Renema et al., 2008) and might be of particular importance to the diversity of elasmobranchs. For example, one of the world's least recorded carcharhinids, the smoothtooth blacktip shark *Carcharhinus leiodon*, is found in the Arabian/Persian Gulf (hereafter referred to as The Gulf) (Moore, White, Ward, Naylor, & Peirce, 2011). Furthermore, many of the shark taxa in the Arabian region are genetically distinct from their closest relatives in neighboring areas (Naylor et al., 2012) and the wider Indo‐Pacific region (e.g., Corrigan et al., 2017; Delser et al., 2016; Haseli, Malek, & Palm, 2010; Naylor et al., 2012; Vignaud, Maynard, et al., 2014; Vignaud, Mourier, et al., 2014; White, Last, Naylor, Jensen, & Caira, 2010). This distinctiveness might have been enhanced by the geological event that resulted in the closure of the Tethys Sea, a major seaway connecting the Atlantic and the Indian Ocean via the Mediterranean Sea and The Gulf (Lambeck, 1996 *)*. During this event, approximately 23–15 million years ago, small isolated water pools formed along The Gulf's seafloor, which are thought to have had an important effect on the origin, dispersal, and speciation of several elasmobranch groups (Last, Matsumoto, & Moore, 2012; Musick, Harbin, & Compagno, 2004).

Population genetic studies of sharks in the water bodies surrounding the Arabian Peninsula are scarce (Jabado & Spaet, 2017; Spaet, Thorrold, & Berumen, 2011), and so far, only one study has examined the population structure in four species of requiem sharks (Spaet, Jabado, Henderson, Moore, & Berumen, 2015). Given the limited data available on sharks in the region (Jabado & Spaet, 2017; Spaet, Cochran, & Berumen, 2011; Spaet, Thorrold, et al., 2011), increasing evidence of depleted shark populations (Clarke, Lea, & Ormond, 2013; Henderson, McIlwain, Al‐Oufi, & Al‐Sheili, 2007; Spaet, Nanninga, & Berumen, 2016), and alarming reports of local fishermen revealing declines in shark abundance of up to 80% (Jabado, Al Ghais, Hamza, & Henderson, 2015; Almojil, 2016), there is an urgent need to provide the basic science required for the conservation of these animals. Here, we used microsatellite markers to investigate the population structure of two regionally exploited (Henderson et al., 2007; Jabado & Spaet, 2017; Spaet & Berumen, 2015) shark species, the spot‐tail shark *Carcharhinus sorrah* and the blacktip shark *C. limbatus*.

*Carcharhinus sorrah* and *C. limbatus* are requiem sharks that reach a maximum total length of 160 and 250 cm, respectively. Throughout the Indo‐west Pacific, they generally occur along continental and insular shelves, over coral reefs and muddy bottoms (Ebert, Fowler, & Compagno, 2013). Based on the International Union for Conservation of Nature (IUCN) Red List criteria, both species are listed as Near Threatened globally (Burgess & Branstetter, 2009; Pillans, Stevens, & White, 2009) and as Vulnerable regionally (Jabado et al., 2017).

*Carcharhinus sorrah* has been shown to exhibit a significant genetic structure over stretches of deep water (Giles et al., 2014). Based on mitochondrial ND2 sequences, substantial genetic divergence was found between individuals from the Timor Sea/Gulf of Carpentaria and those from Borneo, the South China Sea, Thailand, and India (Naylor et al., 2012). Genetic studies of *C. sorrah* across northern Australia, in contrast, have suggested a panmictic population structure (Lavery & Shaklee, 1989; Ovenden, Kashiwagi, Broderick, Giles, & Salini, 2009). Although the species can move long distances (>1,000 km), almost 50% of tagged individuals in a tracking study were recaptured within 50 km of their tagging site (Stevens, West, & McLoughlin, 2000). This suggests that movement of most individuals is limited, probably resulting in little mixing between sites.

*Carcharhinus limbatus* is known to travel distances of over 2,000 km, with movements being influenced by seasonal changes in surface water temperatures (Kohler & Turner, 2001). The species uses shallow coastal waters as nurseries where juveniles spend the first months of their lives (Heupel & Simpfendorfer, 2002; Simpfendorfer & Milward, 1993). Evidence of genetic structure was found between nurseries in North America, the Gulf of Mexico, and the Caribbean (Keeney, Heupel, Hueter, & Heist, 2005). Females were hence suggested to disperse nonrandomly and to exhibit philopatric behavior (Keeney, Heupel, Hueter, & Heist, 2003). Pronounced structuring was detected between African (KwaZulu‐Natal and Sierra Leone) and Indo‐Pacific populations and those of the eastern Atlantic based on mitochondrial DNA (mtDNA) (Keeney & Heist, 2006). However, this analysis did not include any South American populations, which were tested later and revealed that *C. limbatus* from northern Brazil is genetically distinct from the previously studied populations (Sodré et al., 2012). The aim of this study was to unravel patterns of connectivity among stocks of these two commercially exploited species along the coasts of the Arabian Peninsula, Pakistan, and KwaZulu‐Natal, South Africa (hereafter referred to as South Africa), to facilitate regional conservation and management.

## 2 MATERIAL AND METHODS

### 2.1 Sample collection and laboratory procedures

**Figure 1:**
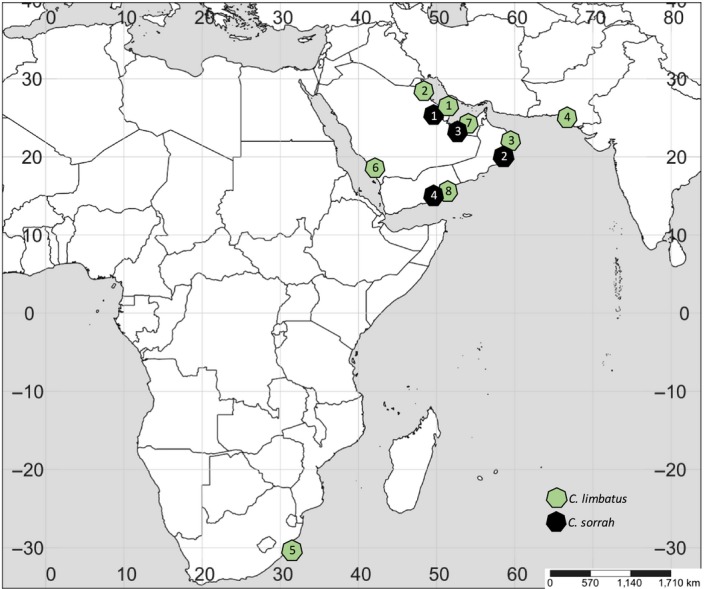
Sample locations for *Carcharhinus sorrah* and *C. limbatus*. Numbers correspond to landing site locations in Table 1

Fin clips or gill slit samples of *C. sorrah* were obtained from local landing sites in Bahrain, Oman, the UAE, and Yemen and of *C. limbatus* from Bahrain, Kuwait, Oman, Pakistan, Saudi Arabia (Red Sea), South Africa, the UAE, and Yemen (Figure 1, Table 1 **)**. Samples from South Africa originated from sharks caught in mesh nets as part of a bather protection program (Dudley & Cliff, 1993). All samples were preserved in 96% ethanol.

**Table 1:** Landing sites sampled between May 2011 and July 2013 and respective sample sizes by country. Number in brackets corresponds to sampling locations in Figure 1

Country	Landing site	Sample size
*C. sorrah*		Total: 327
Bahrain	Al Manama (1)	51
Oman	Barka, Muscat, Qurayat, Kholouf, and Mirbat (2)	87
UAE	Dubai, Abu Dhabi, and Ras Al Khaima (3)	96
Yemen	Hadhramout and Qusayer (4)	93
*C. limbatus*		Total: 525
Bahrain	Al Manama (1)	12
Kuwait	Sharq and Fahaheel (2)	12
Oman	Barka, Muscat, Qurayat, Kholouf, and Mirbat (3)	90
Pakistan	Karachi (4)	57
SAF	KwaZulu‐Natal^a^ (5)	93
SAR	Jeddah (6)	91
UAE	Dubai, Abu Dhabi, and Ras Al Khaima (7)	85
Yemen	Hadhramout and Qusayer (8)	85

Notes

SAF: South Africa; SAR: Saudi Arabia.

a Samples from KwaZulu‐Natal originated from sharks caught in large‐mesh nets, which were deployed off KwaZulu‐Natal as part of a bather protection program (Dudley & Cliff, 1993).

Sharks landed along the coasts of the Arabian Peninsula were assumed to originate from fleets operating within a restricted range. To ensure that the origin of the collected specimens was accurately represented by their landing sites, fishermen were asked to report their approximate fishing grounds and trip lengths. Moreover, observations on boat length, design, and engine power were made whenever possible to verify the reported fishing range. Not included in the study were samples originating from boats with offshore operating capacities, that is, medium‐sized boats (>15–18 feet), characterized by a deep‐V hull design, portable fuel gallons, and an engine >400 horse power. Tissue sampling was randomized by collecting no more than ten samples of each species on the same day. The only exception was Pakistan where landings of *C. limbatus* only occurred on the last day of fieldwork (*n* = 57). A breakdown of sex and size composition for all samples is available in Supporting Information Table S1. In addition, samples of 18 pregnant *C. sorrah* females with a total of 78 pups were collected from Deira fish market, Dubai, the UAE. These samples were not included in the population structure analysis but were instead used to detect null alleles by checking for genotype mismatches (i.e., genotypes that do not share a common allele) between pups and their known mothers (Marshall, Slate, Kruuk, & Pemberton, 1998).

Total genomic DNA from Red Sea samples was extracted following the protocol described in Spaet et al. (2015). DNA from all other samples was extracted using an adjusted glass milk protocol (Boom et al., 1990). Samples were incubated overnight in lysis solution (10 mM Tris‐HCL (pH 8.0) and 1 mM EDTA, 1% SDS, and 50 μg/ml proteinase K. Samples were then centrifuged, the supernatant was transferred to a new tube with sodium iodide (NaI), and 10 μl of glass milk solution were added. The DNA was washed with 500 μl of a solution that comprised of 100 mM NaCl, 1 mM EDTA and 10 mM Tris and 50% ethanol). Pellets were dried and then washed with 500 μl of 1× TE solution (500 μl of 10 mM Tris, 100 μl of EDTA, and 49.4 ml of distilled water). The extracted DNA was eluted into a new tube in 1× TE. Finally, the quality and quantity of the extracted DNA was checked from a random subset of the extracted samples using a NanoDrop spectrophotometer, ND‐1000 Serial 7749, device (Thermo Scientific, UK).

### 2.2 Amplification and genotyping

For *C. sorrah*, DNA amplification was performed using 15 species‐specific polymorphic loci (Supporting Information Table S2a,b). For *C. limbatus*, 11 loci were used of which ten were species specific (Supporting Information Table S2c,d) (Almojil et al., 2016). Amplification was performed using the Qiagen multiplex PCR kit (Qiagen, Redwood, California). Multiplex PCRs were carried out in a total volume of 10 μl, containing approximately 20 ng of genomic DNA, 5 μl multiplex master mix solution, 1 μl primer mix, and 2 μl of RNase‐free water. For each species, primers were organized into two sets of primer mix (Supporting Information Table S2a–d). PCR cycles were run using the following cycling conditions: initial denaturation of 5 min at 95°C, followed by 30 cycles of 30 s at 95°C, 30 s at 60°C, and 1 min at 72°C. For each PCR plate, two wells containing the whole PCR mix but no DNA were used as a negative control for each run. PCR products were diluted in autoclaved water into 1:15 dilutions. Subsequently, 0.7 μl of the diluted product was transferred to a MicroAmp plate containing 10 μl of formamide and GeneScan (Liz 500) ladder (Life Technologies, Cheshire). The MicroAmp plate was run on a 3730XL DNA sequencer (Thermo Fisher Scientific). To avoid any plate‐specific bias due to possible effects of PCR performance errors, individual samples originating from the same location were randomized by distributing them across different plates for the amplification and genotyping process.

### 2.3 Data analysis

#### 2.3.1 Genetic diversity

Alleles were scored using the program GENEMAPPER (v3.7; Applied Biosystems). All samples were scored blindly, without knowledge of the sampling location to avoid any unintentional bias. To account for genotyping errors, we used standard likelihood‐based methods as implemented in the program GENEPOP (v3.3; Raymond & Rousset, 1995). In addition, we determined the number of mismatches between reference genotypes and regenotyped replicates (Bonin et al., 2004; DeWoody, Nason, & Hipkins, 2006; Hoffman & Amos, 2005; Pompanon, Bonin, Bellemain, & Taberlet, 2005). Furthermore, we checked for Mendelian‐inconsistent errors by determining mismatch error rates between mother and pup samples. Error rates were calculated for each locus by dividing the number of mismatched genotypes by the total number of genotypes (Marshall et al., 1998). The latter analysis was only performed for *C. sorrah* due to the unavailability of matched mother and pup samples for *C. limbatus*.

Concordance with Hardy–Weinberg equilibrium (HWE) and a test for linkage disequilibrium were performed using ARLEQUIN (v3.5; Excoffier & Lischer, 2010) and GENEPOP. Conformance of expectations of HWE for each locus and population was tested using the exact test with 1,000 batches and 10,000 iterations per batch and a significance level set at 0.05. All multiple comparison *p* values were corrected with sequential Bonferroni's adjustment in R (v.2.7.2; R Team 2015). Allelic richness was determined in GENALEX (v6; Peakall & Smouse, 2006) using the rarefaction method, which accounts for differences in sample size.

#### 2.3.2 Population structure

The degree of genetic differentiation among sampling sites and locations was estimated using corrected pairwise *F*_ST_ measured in GenoDive (v2.0; Meirmans & Van Tienderen, 2004). Pairwise *F*_ST_ was tested for significance at level 0.05 with 10,000 permutations. Multiple comparison *p* values were corrected with false discovery rate (FDR) adjustment in R (v.2.7.2; R Team 2015). Neighbor‐joining trees using pairwise *F*_ST_ between different locations were constructed using the adegenet package in R (Jombart, 2008). STRUCTURE (v2.3.3; Falush, Stephens, & Pritchard, 2007) was used to estimate the number of genetically differentiated clusters (*K*) for each species. Five runs were generated per *K* value tested, with *K* ranging from 1 to 10. Simulations were run with a burn‐in period of 100,000 steps, followed by a Markov chain Monte Carlo (MCMC) iteration of 100,000 steps. The length of the burn‐in period was verified by ensuring that the Ln *P(D*) and the likelihood of the runs had stabilized. A correlated allele frequency model was used with sampling site as location prior and admixture were assumed, as recommended when population structure is likely to be subtle (Falush et al., 2007; Hubisz, Falush, Stephens, & Pritchard, 2009). The mean of Ln *P(D*), which is a model choice criterion to select for the true value of *K* (Pritchard, Stephens, & Donnelly, 2000), was selected and plotted for each *K* using our own script in R (v.2.7.2; R Team 2015). The script was designed to average the log likelihood [Ln P(D)] of each value of *K* to indicate the estimated probability that the number of *K* is the most probable to fit the data.

Discriminant analysis of principal components (DAPC) was performed using the R package adegenet. DAPC has the advantage of using predefined clusters identified using a clustering algorithm. It optimizes variance among these clusters, while minimizing the variance within them to illustrate differences between them (Jombart, Devillard, & Balloux, 2010). After dividing individuals into clusters, a membership probability plot was constructed. Additionally, a scatterplot was constructed based on 100 PCs. The retained number of PCs was chosen using cross‐validation (Jombart & Collins, 2015). In the resultant graph, each individual is represented by a dot, which allows clear visualization of estimated proximities between populations inside the data space.

Isolation by distance (IBD) was tested using a Mantel test implemented in the R package adegenet. The geographic distance between locations was measured in kilometers using Google Maps (© DigitalGlobe 2015). Measures of geographic distance were taken as straight lines drawn along the coast and then plotted against genetic distances (corrected *F*_ST_).

To test for possible effects of sex‐biased dispersal on partitioning genetic variation, a corrected assignment index (*AI* ^c^) (Paetkau, Calvert, Stirling, & Strobeck, 1995) was computed for each individual in GENALEX. Negative *AI* ^c^ values characterize individuals with a lower‐than‐average probability of being born locally; hence, the sex showing on average more negative values is considered the dispersing sex. To evaluate the potential differences in dispersal between sexes, the difference in *AI* ^c^ values between males and females was tested using a Wilcoxon's rank‐sum test.

#### 2.3.3 Demographic history

*BOTTLENECK* (v.1.2.02; Piry, Luikart, & Cornuet, 1999) was used to test for heterozygosity excess as evidence of a recent reduction in effective population size (*N*_e_), under three possible mutation models: the infinite allele model (IAM), the single‐step mutation model (SMM), and the two‐phase model (TPM).

## 3 RESULTS

### 3.1 Genetic diversity and population structure

#### 3.1.1 *Carcharhinus sorrah*

Summary statistics averaged across all loci indicated similar levels of genetic diversity across all four sampling locations (Table 2). All locations showed relatively high levels of heterozygosity, with observed values (*H*_o_ ± *SE*) ranging from 0.64 ± 0.16 in Bahrain to 0.69 ± 0.15 in Yemen. Allelic richness ranged from 3.5 ± 0.4 (Bahrain) to 3.9 ± 0.4 (the UAE and Yemen). The value of *F*_IS_, an inbreeding coefficient measure that calculates the proportion of the variance in the subpopulation contained in an individual (Raymond & Rousset, 1995), was small at all locations ranging from −0.01 ± 0.01 (Oman) to 0.01 ± 0.02 (the UAE) (Table 2).

**Table 2:** Genetic diversity indices for *C. sorrah* and *C. limbatus*, based on microsatellite loci averaged for each location across all loci

	*N*	A	R	*H*_O_	*H*_E_	*F*_IS_
*C. sorrah*						
Bahrain	51	7.8 ± 0.8	3.5 ± 0.4	0.64 ± 0.16	0.66 ± 0.17	0.004 ± 0.01
Oman	87	9.2 ± 0.9	3.8 ± 0.5	0.68 ± 0.17	0.67 ± 0.16	−0.01 ± 0.01
UAE	96	8.8 ± 0.8	3.9 ± 0.4	0.68 ± 0.14	0.69 ± 0.13	0.01 ± 0.02
Yemen	93	8.3 ± 0.7	3.9 ± 0.4	0.69 ± 0.15	0.69 ± 0.14	−0.001 ± 0.01
*C. limbatus*						
Bahrain	12	6.8 ± 0.6	4.3 ± 0.5	0.62 ± 0.05	0.7 ± 0.04	0.13 ± 0.04
Kuwait	12	6.6 ± 0.5	4.3 ± 0.4	0.67 ± 0.04	0.7 ± 0.05	0.03 ± 0.03
Oman	90	10.4 ± 1.1	4 ± 0.5	0.69 ± 0.04	0.7 ± 0.03	0.023 ± 0.02
Pakistan	57	8.8 ± 0.7	4 ± 0.4	0.64 ± 0.05	0.71 ± 0.04	0.09 ± 0.05
SAF	93	9.5 ± 0.9	4.2 ± 0.4	0.69 ± 0.03	0.71 ± 0.03	0.02 ± 0.01
SAR	91	9.5 ± 0.8	4 ± 0.4	0.73 ± 0.04	0.71 ± 0.04	− 0.03 ± 0.02
UAE	85	10.6 ± 1.3	3.9 ± 0.4	0.68 ± 0.04	0.71 ± 0.03	0.04 ± 0.03
Yemen	85	9.6 ± 0.8	3.6 ± 0.3	0.66 ± 0.04	0.68 ± 0.4	0.02 ± 0.04

Notes. *N*: number of samples; A: number of alleles; R: allelic richness; *H*_O_: observed heterozygosity; *H*_E_: expected heterozygosity; *F*_IS_: inbreeding coefficient. (Results are reported as mean ± *SD*) SAF: South Africa; SAR: Saudi Arabia.

Null allele frequencies were low at most loci (Supporting Information Table S3), with only two loci showing high null allele frequencies [CS40 (6%), CS55 (5%)] (Supporting Information Table S3). Mismatches between reference genotypes and regenotyped replicates were also low, with only one locus (CS55) displaying a high rate (≥5%) of genotyping error (Supporting Information Table S4), due to incorrect allele scoring. Two loci (CS40, CS55) showed higher rates of genotypic mismatch between mothers (*n* = 18) and pups (*n* = 78) than the rest of loci (Supporting Information Table S5). These two loci were consistent in their unreliability across the genotyping error tests and thus were considered unreliable and were excluded from further analysis.

Pairwise *F*_ST_ values were low but mostly significant (Table 3). Samples from Bahrain showed higher and significant differentiation from all other locations (*F*_ST_ = 0.03, *p *<* *0.001 for all comparisons) (Table 3). The probability support produced by STRUCTURE for a range of Ks (1–10) was highest for *K* = 1, indicating a single population (Supporting Information Figure S1a).

**Table 3:** Pairwise corrected *F*_ST_ values for *C. sorrah* for all sampling locations calculated in GenoDive (Meirmans & Van Tienderen, 2004)

	Bahrain	Oman	UAE
Oman	0.03**	–	
UAE	0.03**	0.01**	–
Yemen	0.03**	0.02**	0.005

Significant: *p *<* *0.05* and highly significant: *p *<* *0.001**.

**Figure 2:**
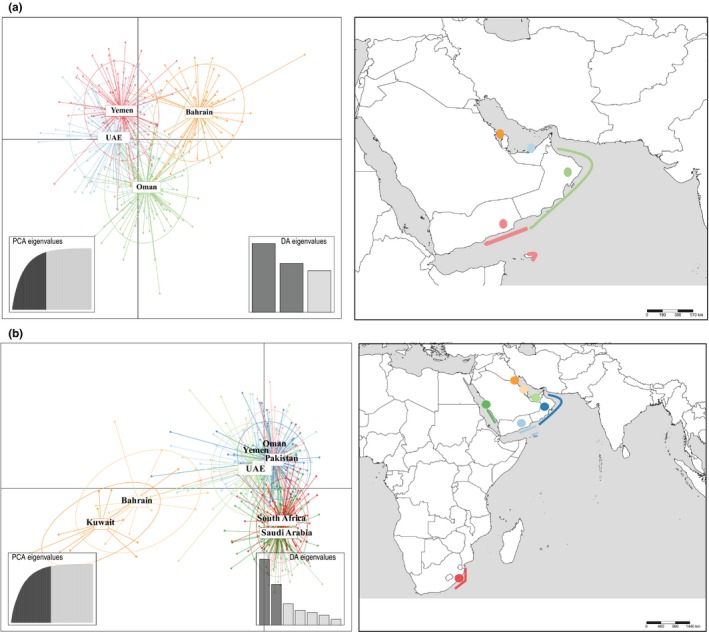
Discriminant analysis of principal component (DAPC) scatterplot for (a) *C. sorrah*, and (b) *C. limbatus*, based on the two‐first discriminate functions. Dots represent individuals from sampling locations illustrated on the map. Inertia ellipses center on the mean for each location inferred from the sampling points. Interconnected ellipses and shared dots within the graph space indicate contemporary gene flow

The DAPC scatterplot supported weak fine‐scale genetic differentiation into three groups. The first group comprises all samples from Bahrain, the second from the UAE and Yemen, and the third from Oman (Figure 2a). A neighbor‐joining tree also illustrated limited gene flow between Bahrain and all other locations (*F*_ST_ = 0.01, *p *<* *0.001) (Supporting Information Figure S2). A Mantel test indicated no significant IBD pattern (*p *=* *0.622). All pairwise comparisons involving Bahrain showed high genetic distance, irrespective of geographic distance (data not shown).

#### 3.1.2 *Carcharhinus limbatus*

Summary statistics averaged across all loci indicated relatively high levels of heterozygosity across all sampling locations (Table 2). Observed heterozygosity was highest for the Saudi Arabian Red Sea (*H*_O_ = 0.73 ± 0.04) and lowest for Bahrain (*H*_O_ = 0.62 ± 0.05). Allelic richness did not greatly differ between sampling locations, ranging from 3.6 ± 0.3 (Yemen) to 4.3 ± 0.5 (Bahrain) (Table 2).

Allele frequencies were low at most loci (Supporting Information Table S6). Only three loci showed high null allele frequencies [AC 60 (12%), AG 2 (8%), AC 17 (8%)] (Supporting Information Table S6). All loci displaying a frequency of null alleles ≥5% were considered unreliable and thus excluded from further analysis.

Numbers of mismatches between reference genotypes and regenotyped replicates were also low (Supporting Information Table S7), with only one locus (AC 17) showing a high rate of genotyping error (≥5%), caused by an allele scoring error. High genotyping error in other markers (AC 60, AG 2) was attributed to failure of amplification. These loci also deviated from HWE, suggesting that failure of amplification might be caused by allele dropout. These loci were hence excluded from further analysis.

Pairwise *F*_ST_ values were mostly low but significant (Table 4). However, samples from Bahrain and Kuwait showed low differentiation from each other but were highly differentiated from all other locations (*F*_ST_ = 0.13–0.19, *p *<* *0.001) (Table 4). The probability support produced by STRUCTURE for a range of Ks (1–10) was highest for *K* = 1, indicating a single population (Supporting Information Figure S1b).

**Table 4:** Pairwise corrected *F*_ST_ values for *C. limbatus* for all sampling locations calculated in GenoDive (Meirmans & Van Tienderen, 2004)

	Bahrain	Kuwait	Oman	Pakistan	SAF	SAR	UAE
Kuwait	−0.13^(ns)^	–					
Oman	0.15**	0.18**	–				
Pakistan	0.14**	0.16**	0.01**	–			
SAF	0.14**	0.17**	0.02**	0.03**	–		
SAR	0.16**	0.19**	0.04**	0.04**	0.01**	–	
UAE	0.13**	0.16**	0.02**	0.01**	0.03**	0.03**	–
Yemen	0.15**	0.17**	0.01**	0.01*	0.02**	0.03**	0.01**

*Notes.* SAF: South Africa; SAR: Saudi Arabia.

Significant: *p *<* *0.05* and highly significant: *p *<* *0.001**.

The DAPC scatterplot also supported population subdivision between three groups. The first group comprises all samples from Bahrain and Kuwait, the second from Oman, Pakistan, the UAE and Yemen, and the third from South Africa and the Saudi Arabian Red Sea (Supporting Information Figure 2b). While the second and third groups showed fine‐scale structuring, samples from Bahrain and Kuwait were highly differentiated from all other locations. This finding was further supported by a neighbor‐joining tree, illustrating limited gene flow between Bahrain and Kuwait and all other locations (*F*_ST_ = 0.01, *p *<* *0.001) (Supporting Information Figure S3). A Mantel test indicated no significant isolation by distance (IBD) pattern (*p *=* *0.455). Yet, all pairwise measures involving Bahrain and Kuwait showed high genetic distance, irrespective of geographic distance (data not shown).

### 3.2 Sex‐biased dispersal

**Figure 3:**
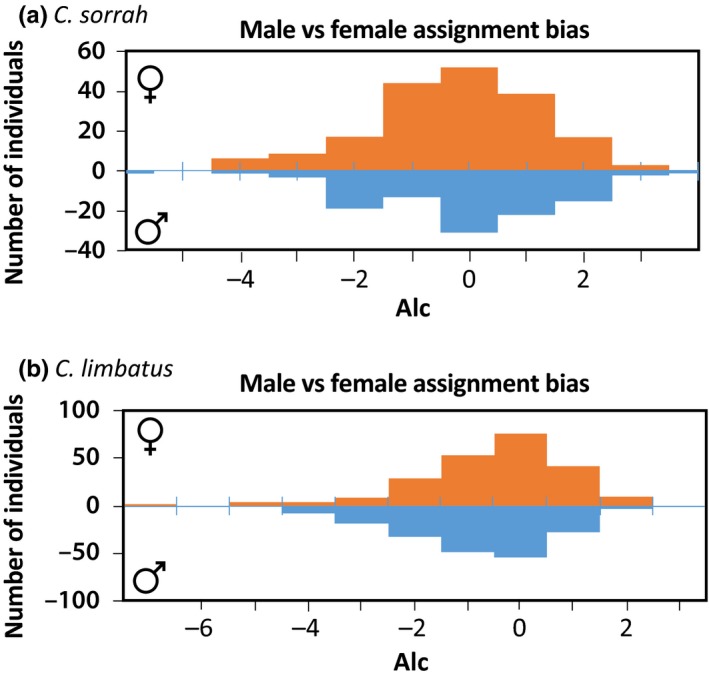
Frequency distribution of the corrected assignment index (*AIc*) for females (orange bars) and males (blue bars) *C. sorrah* (a), and *C. limbatus* (b) across all sampling locations

The frequency distribution of *AI*_c_ for *C. sorrah* differed slightly among sexes (Figure 3a). Males had more positive values, while females had more negative values. Mean *AI*_*c*_ values were lower for females (−0.07 ± 0.2 *cf*. 0.12 ± 0.2 (±*SE*)) (Figure 3a), yet a Wilcoxon's rank‐sum test between sexes was not significant (*W* = 17,484, *p *=* *1) (Supporting Information Figure S4a).

The frequency distribution of *AI*_c_ for *C. limbatus* was similar among sexes (Figure 3b); however, the mean assignment bias for females showed significantly higher *AI*_c_ values than males (0.5 ± 0.1 vs. −0.02 ± 0.1 (*SE*); *W* = 18,951, *p *=* *0.008, Wilcoxon's rank‐sum test) (Supporting Information Figure S4b).

### 3.3 Demographic history

Heterozygosity excess differed under the BOTTLENECK mutation models (IAM, TPM, and SMM) in both species (Supporting Information Tables S8 and S9). Of the four populations analyzed for evidence of a bottleneck in *C. sorrah*, the IAM showed evidence of heterozygosity excess for the UAE and Bahrain populations. Under the SMM and TPM, all populations showed evidence of heterozygosity excess.

Of the eight populations analyzed for evidence of a bottleneck in *C. limbatus*, the IAM model showed no evidence of heterozygosity excess for all but the Saudi Arabian Red Sea and the South African populations. The TPM model supported evidence of heterozygosity excess for all but the Kuwait and Pakistan populations, while the SMM model showed evidence for all populations.

## 4 DISCUSSION

This study presents a regional analysis of the genetic population structure of two potentially overexploited shark species (Jabado et al., 2015; Spaet & Berumen, 2015; Spaet et al., 2016) along the coasts of the Arabian Peninsula, Pakistan, and South Africa. Overall, our findings support three populations for both species. Population subdivision was supported by pairwise *F*_ST_ and DAPC, but not by STRUCTURE. The failure of STRUCTURE to identify genetic heterogeneity might be attributed to (a) a variation in sample size among sampling locations (*n* = 51–96) (Kalinowski, 2011; Puechmaille, 2016) or (b) the complexity and discontinuity of the data space (e.g., multimodality) (François & Durand, 2010; Gilks, 2005) or (c) limited genetic differentiation among populations (Latch, Dharmarajan, Glaubitz, & Rhodes, 2006). In situations of weak genetic differentiation, DAPC has proven to be a powerful tool in detecting fine‐scale structure (Novembre et al., 2008; O'Connor et al., 2015; Patterson, Price, & Reich, 2006). We hence believe that for our dataset, maximizing variance between predefined clusters, while minimizing variance within clusters as employed by DAPC (Jombart et al., 2010), was the more sensitive and therefore more appropriate approach to illustrate the observed fine‐scale differences.

*F*_ST_ values supporting the population structure in both *C. sorrah* and *C. limbatus* were mostly low, yet significantly different from zero. Low but significant genetic divergence is a common finding in genetic population studies of marine organisms (reviewed in Ward, Woodwark, & Skibinski, 1994). In sharks, this pattern has been reported in a variety of species (e.g., Keeney et al., 2005; Nance, Klimley, Galván‐Magaña, Martínez‐Ortíz, & Marko, 2011; Portnoy et al., 2010; Portnoy et al., 2014; Schmidt et al., 2009; Tillett et al., 2012; Vignaud, Maynard, et al., 2014). Past studies on coastal shark populations suggest that behavioral traits such as residency and return migration (e.g., philopatry and site fidelity) can result in fine population structure, such as the one observed in our study (Chapman, Feldheim, Papastamatiou, & Hueter, 2015; Hueter, Heupel, Heist, & Keeney, 2005). Findings from other studies across different taxa at different spatial and temporal scales have also linked fidelity behavior to geographic structuring (Aykanat et al., 2015; Chesser, 1991; Knutsen et al., 2011; Miller et al., 2010; Schaefer, Bergman, & Luttich, 2000; Schmitt et al., 2014; Storz, 1999; Van Beest, Vander Wal, Stronen, Paquet, & Brook, 2013). High levels of philopatry can lead to demographic isolation (Bose et al., 2017; Marescot, Forrester, Casady, & Wittmer, 2015), resulting in small‐scale differences in population growth.

In sharks, residency, site fidelity, and philopatry have been reported in a diverse range of species (reviewed in Chapman et al., 2015). Residency has previously been observed in *C. sorrah* (Knip, Heupel, & Simpfendorfer, 2012a,b), but there is no evidence for natal philopatry in this species (Chapman et al., 2015). Seasonal residency, regional philopatry, and site fidelity have been reported for *C. limbatus* (Chapman et al., 2015; Gledhill et al., 2015; Heupel & Simpfendorfer, 2002; Keeney et al., 2005). Fine‐scale population structure owing to residential behavior and possibly natal philopatry has been suggested for the blacktip reef shark *C. melanopterus* (Mourier, Mills, & Planes, 2013; Papastamatiou, Caselle, Friedlander, & Lowe, 2009; Papastamatiou, Friedlander, Caselle, & Lowe, 2010) and *C. limbatus* in coastal habitats of the Gulf of Mexico (Heupel & Simpfendorfer, 2002; Hueter et al., 2005). There, *C. limbatus* show seasonal residency of their natal sites for at least their first year and leave to avoid thermal stress when temperatures decline (Heupel & Simpfendorfer, 2002; Hueter et al., 2005).

Philopatry of *C. sorrah* and *C. limbatus* around the Arabian Peninsula was not supported by Spaet et al. (2015) based on nuclear and mtDNA. Yet, mtDNA variation observed by Spaet et al. (2015) might have been insufficient to detect the possible genetic heterogeneity. Past studies detecting philopatry in *C. limbatus* either showed higher mtDNA haplotype and nucleotide diversity (Keeney et al., 2003) than that observed in Spaet et al. (2015) or focused their sampling on neonates collected from nursery grounds (Hueter et al., 2005; Keeney et al., 2003). Failure to detect possible philopatry due to low mtDNA diversity has previously been observed in sharks (Martin, Naylor, & Palumbi, 1992; Portnoy et al., 2016), as well as in yellowfin tuna, *Thunnus albacares* (Ely et al., 2005).

We tested for evidence of sex‐biased dispersal of *C. sorrah* and *C. limbatus* using *AI*_*c*_. While *C. sorrah* females showed on average more negative values, this result was not significant, indicating that dispersal in this species is likely not sex‐biased. By contrast, the mean assignment bias for *C. limbatus* females showed significantly higher *AI*_*c*_ values than that for males, suggesting that females could be philopatric and males are the dispersing sex. If breeding occurs at specific sites and females are philopatric, low but significant *F*_ST_ values are generated among breeding sites, as male‐mediated gene flow cannot completely remove the structure generated by female philopatry. This is because internal population dynamics can still be generated when populations are connected by male dispersal only, as adult females might form discrete demographic aggregations.

IBD results for both species imply that the observed structure is unlikely a result of geographic distance. For example, despite the vast distance between South Africa and Saudi Arabia (~10,000 km), *C. limbatus* from these two locations were grouped together by DAPC. A possible explanation is the presence of contemporary male‐mediated gene flow connecting these two locations. Long‐distance male‐mediated gene flow was also documented in the sandbar shark *C. plumbeus* (~8,000 km between East Australia and Hawaii) (Portnoy et al., 2010), Lemon shark (*Negaprion ssp*) (Schultz et al., 2008), and across ocean basins in the shortfin mako shark *Isurus oxyrinchus* (Schrey & Heist, 2003). The movement of *C. limbatus* males between South Africa and Saudi Arabia could be facilitated by favorable nearshore sea surface temperatures along the entire East African coast, unlike the West African coast where the cold Benguela Current in the south would be a barrier to the movement of *C. limbatus* between South Africa and the populations of the northwestern Atlantic. Another interesting observation is that *C. sorrah* individuals from Bahrain and *C. limbatus* individuals from Bahrain and Kuwait showed the greatest genetic distance to all other locations. The distinctiveness of samples from these two locations might have been established through founder effects during the Tethys Sea closure. The Gulf was almost entirely drained 18,000 years ago as a result of a drop in sea level (Sheppard, Price, & Roberts, 1992). During this period, changes in The Gulf's biodiversity assemblage through genetic differentiation (Hoolihan, Premanandh, D'Aloia‐Palmieri, & Benzie, 2004) and fish speciation (Last et al., 2012) might have occurred in remaining isolated pools of water (Hoolihan et al., 2004; Last et al., 2012). Even with contemporary gene flow between the northern and southern parts of The Gulf, the exchange might have been limited by colder sea surface temperatures and strong seasonal fluctuations in temperature inherent to the northern and central parts of this ocean basin (Sheppard et al., 1992) (Supporting Information Table S10), potentially discouraging sharks from moving to colder areas of The Gulf. Annual sea surface temperatures for both Bahrain and Kuwait are on average lower than temperatures for Oman, the UAE, and Yemen (24°C ± 1.8 and 25.9°C ± 1.7 *cf*. 27.5°C ± 0.9, 27.5°C ± 1.4 and 27.5°C ± 0.5). Furthermore, the seasonal variation in water temperature in The Gulf is largest for Bahrain and Kuwait (30°C and 37°C *cf*. 9°C, 22°C and 3°C for Oman, the UAE and Yemen, respectively), indicating that temperatures are less stable at Bahrain and Kuwait (Supporting Information Table S10). Elsewhere, changes in sea surface temperature have been shown to influence the movement of sharks (Keeney & Heist, 2006). In particular, the offspring of *C. limbatus* migrates from nursery grounds to offshore wintering grounds when temperatures drop below 21°C (Castro, 1996). This supports our hypothesis of colder sea surface temperatures potentially limiting gene flow between warmer and colder areas of The Gulf.

Based on our findings, populations from Bahrain and Kuwait have possibly experienced founder effects and population structuring as recent as 10,000 years ago. Moreover, bottleneck analysis under the SMM model, which is the most appropriate model for microsatellite analyses (Piry et al., 1999), suggested a significant recent reduction in the effective population size, showing significant excess of heterozygosity for all populations in both species. Given the long generation time of our study animals [4.3 and 10 years for *C. sorrah* and *C. limbatus*, respectively (Cortés, 2002)], their populations have most likely not reached an equilibrium state yet.

Future research to understand the role of philopatric behavior in generating fine‐scale structure in shark populations (Momigliano et al., 2017; Pazmiño et al., 2018; Portnoy et al., 2015) in the Arabian region is warranted. Particular focus should be placed on a comparison of geographic scales of heterogeneity partition produced by neutral (both microsatellite and mtDNA) vs. non‐neutral markers. In addition, it would be interesting to assess whether genetic heterogeneity is structured at non‐neutral markers among nursery grounds.

## 5 CONCLUSION

Findings of this study have contributed to our understanding of the population structure of *C. sorrah* and *C. limbatus* along the coasts of the Arabian Peninsula, Pakistan, and South Africa. Based on the nuclear markers used in this study, we suggest that both *C. sorrah* and *C. limbatus* exhibit populations subdividing the area into three groups. In *C. sorrah,* the first group comprises all samples from Bahrain, the second from the UAE and Yemen, and the third from Oman. In *C. limbatus*, the first group comprises samples from Bahrain and Kuwait, the second from Oman, Pakistan, the UAE, and Yemen, and the third from South Africa and the Saudi Arabian Red Sea. The generally weak population structure observed in this study may possibly be due to the effect of sex‐biased dispersal (i.e., through site fidelity or philopatry), which could promote population closure on finer geographic scales. The distinctiveness of all samples from Bahrain and Kuwait from all other sampling locations could be the result of founder effects during the Tethys Sea closure. Overall, our study suggests that conservationists and resource managers should treat each of the three mentioned groups as separate conservation units.

## CONFLICT OF INTEREST

None declared.

## AUTHOR CONTRIBUTIONS

Dareen Almojil designed the study, collected samples for *C. limbatus* and *C. sorrah* from Kuwait, Bahrain, UAE, Oman, Yemen, and Pakistan, analyzed and interpreted the data, and wrote the manuscript. Julia Spaet provided tissue samples for *C. limbatus* from Saudi Arabia and critically reviewed and edited the manuscript. Geremy Cliff provided tissue samples for *C. limbatus* from South Africa. All authors approved the final version of the manuscript.

## DATA ACCESSIBILITY

The entire dataset used in this study has been deposited in the Dryad Repository, https://doi.org/10.5061/dryad.811nr62.
