# Supplementary material for: Weak population structure of the Spot‐tail shark Carcharhinus sorrah and the Blacktip shark C. limbatus along the coasts of the Arabian Peninsula, Pakistan, and South Africa
Source: Ecol Evol. 2018 Aug 29;8(18):9536–49. doi: 10.1002/ece3.4468 (PMC6194305; doi:10.1002/ece3.4468)
Supplement: Supplementary file 1 [file ECE3-8-9536-s001.docx]

**Supplementary Information for:**

Weak population structure of the Spot-tail shark *Carcharhinus sorrah* and the Blacktip shark *C. limbatus* along the coasts of the Arabian Peninsula, Pakistan and South Africa.

| **Table S1** Composition of *C. limbatus* and *C. sorrah* samples collected between May 2011 - July 2013 by country: total sample size (n), Sample size by sex (percentage of n), size range by sex (STL ± S.D.). SAF = South Africa and SAR = Saudi Arabia. | | | |
| --- | --- | --- | --- |
| **Country** | **Sample size** | **Sample size** | **Size range (mean± S.D)** |
| **Bahrain** | 51 | F: 28 (55%) | F: 78 - 144 (100 ± 3) |
|  |  | M: 23 (45%) | M: 79 - 125 (89 ± 1.5) |
| **UAE** | 96 | F: 63 (66%) | F: 98 - 159 (139 ± 1.2) |
|  |  | M: 33 (34%) | M: 100 - 131(120 ± 1) |
| **Oman** | 87 | F: 61 (70%) | F: 77 - 175 (130 ± 3.3) |
|  |  | M: 26 (30%) | M: 79 - 149 (116 ± 2.5) |
| **Yemen** | 93 | F: 52 (56%) | F: 72 - 166 (120 ± 2.8) |
|  |  | M: 41 (44%) | M: 72 - 154 (112 ± 2.2) |
| **Total:** | **327** |  |  |
| **Bahrain** | **12** | F: 6 (50%) | F: 51 - 170 (124 ± 9.3) |
|  |  | M: 6 (50%) | M: 53 - 55 (54 ± 0.004) |
| **Kuwait** | **12** | F: 7 (58%) | F: 51 - 170 (115 ± 0.7) |
|  |  | M: 5 (42%) | M: 58 - 60 (61 ± 0.6) |
| **Oman** | **90** | F: 42 (46%) | F: 67 - 299 (208 ± 2.3) |
|  |  | M: 42 (46%) | M: 71 - 272 (206 ± 1.9) |
| **Pakistan** | **57** | F: 41 (71%) | F: 48 - 256 (154 ± 1.5) |
|  |  | M: 16 (28%) | M: 47 - 189 (73 ± 2.8) |
| **SAF** | **93** | F: 35 (37%) | F: 138 - 244 (202 ± 0.8) |
|  |  | M: 39 (41%) | M: 122 - 231(207 ± 0.6) |
| **SAR** | **91** | F: 46 (50%) | F: 64 - 190 (98 ± 0.6) |
|  |  | M: 44 (48%) | M: 55 - 215 (102 ± 0.8) |
| **the UAE** | **85** | F: 41 (48%) | F: 146 - 242 (199 ± 0.6) |
|  |  | M: 44 (52%) | M: 50 - 232 (141 ± 0.7) |
| **Yemen** | **85** | F: 43 (50%) | F: 67 - 242 (100 ± 1.2) |
|  |  | M: 42 (49%) | M: 68 - 231(86 ± 0.9) |
| **Total:** | **525** |  |  |

| \| **Table S2** Multiplex mixes used in this study, including primer sequence, motif, size range and dye of species-specific loci developed for *C. sorrah* and *C. limbatus*. ^1;2^ indicate primers sourced from the literature: ^1^Keeney & Heist (2003a); ^2^Giresi et al. (2012). \| \| \| \| \| \| --- \| --- \| --- \| --- \| --- \| \| **a): *Carcharhinus sorrah* - Mix 1** \| \| \| \| \| \| **Locus** \| **Primer sequence 5′ → 3′** \| **Motif** \| **Size range** \| **Dye** \| \| CS 8 \| F: CCCAACAACCTTTCCCTCCT  R: TCTTTCACACTTGCTTCACACA \| AC \| 132 - 186 \| 6-FAM \| \| CS 433 \| F: AGTGAGCTGCGGAAGTCATT  R: GGTCTCCCAGCAGAACTCTC \| AT \| 205 - 221 \| 6-FAM \| \| CS 295 \| F: GGGCATCCTCCTTTCCATCT  R: GACTTTTCCCTTCCCGCTTG \| AC \| 207 - 237 \| HEX \| \| CS 102 \| F: TTGCTGCCAGTCCCCTAAG  R: GACAGGTTCGATGGCCAATG \| AG \| 185 - 221 \| NED \| \| CS 104 \| F: GATTGCCATCCCAACATGCT  R: AGCCTCAGGTGATGGTGATT \| AG \| 145 - 167 \| PET \| \| CS 51 \| F: CAATGAAGACACAGGACGCA  R: TCAGAGGTGGGGAGGGATAA \| AC \| 187 - 217 \| PET \|   **b) *Carcharhinus sorrah* - Mix 2** | | | | |
| --- | --- | --- | --- | --- | --- | --- | --- | --- | --- | --- | --- | --- | --- | --- | --- | --- | --- | --- | --- | --- | --- | --- | --- | --- | --- | --- | --- | --- | --- | --- | --- | --- | --- | --- | --- | --- | --- | --- | --- | --- | --- | --- | --- | --- | --- | --- | --- | --- | --- |
| **Locus** | **Primer sequence 5′ → 3′** | **Motif** | **Size range** | **Dye** |
| CS 288 | F: CGCAGGCAAAGGAAGAATGT  R: CCTCTTTCCAGTCCTCAGCA | AC | 133-169 | 6-FAM |
| CS 25 | F: GCACCGCACTTTTGTCTTTG  R: CCCAGAGCCCCAGAACATTA | AC | 184-204 | 6-FAM |
| CS 381 | F: GCATTCCTGAGAGTTGAAAGCT  R: TCATATCACGCCCTCTCACC | AG | 220-248 | 6-FAM |
| CS 179 | F: TGGGGTCTGTGCTGAAGAAT  R:GTATGCGCTGGGATTGCTTT | AG | 146-168 | HEX |
| CS 109 | F: AGCAATCGTGGCAGCATTAC  R: TCCTTCCACCCTTTTCTGCT | AG | 141-171 | NED |
| CS 29 | F: TGGACACACATACATGCCCA  R: GAAGCGATTCAGGAAGGCAG | AC | 241-273 | NED |
| AG 11 | F: TGAGTGGGTGCAGAACAAAC  R: TAGCTGCACTTTGACTGACAC | AG | 115-141 | PET |
| CS 94 | F: TTAGTGTGTCAGGCTCAGCT  R: CCTCCCAACATAGACAGGCA | AG | 150-182 | PET |
| CS 400 | F: CCATGGTCAGCAATGCCTTT  R: GCCTGAATGTTGCAGCTCTT | AG | 218-250 | PET |

| **c) *Carcharhinus limbatus* - Mix 1** | | | | |
| --- | --- | --- | --- | --- |
| **Locus** | **Primer sequence 5′ → 3′** | **Motif** | **Size range** | **Dye** |
| Cli 107^1^ | F: GGATTCACAACACAGGGAAC  R: CTCATTCTTAGTTGCTCTCG | GT | 87-123 | 6-FAM |
| AG 5 | F: TGCAGCAAAACACAGAGTCC  R: TGCTGGGGTTGAACTCTTGA | AG | 170-192 | 6-FAM |
| AC 50 | F: AACCCACATACTCACCCACG  R: TGCTAACCTTCTGACCAGCA | AC | 205-273 | 6-FAM |
| AG 10 | F: CAAGCTCATCCAACAGGCAA  R: ATCCCTCAGTACTGCGCTAG | AG | 198-216 | PET |
| AC 35 | F: TTGCTACCACTGACCTTGCA  R: GTGACTCACCTGCAATAAACCA | AC | 132-156 | NED |

| **d) *Carcharhinus limbatus* - Mix 2** | | | | |
| --- | --- | --- | --- | --- |
| **Locus** | **Primer sequence 5′ → 3′** | **Motif** | **Size range** | **Dye** |
| AC 47 | F: AGAAGGATGAGGCTTTGGTTT  R: ACATCTTCCACAGCTAGGCA | AC | 182-220 | 6-FAM |
| AC 27 | F: CACACGCATGCAAACACAAA  R: CTCTTCCCACAAATCCCAGC | AC | 132-180 | HEX |
| AG 11 | F: TGAGTGGGTGCAGAACAAAC  R: TAGCTGCACTTTGACTGACAC | AG | 117-139 | PET |
| AC 15 | F: GCCACTCAACCCATCTTGTC  R: TGGAGGGAGGGTCAGAGATA | AC | 210-232 | PET |
| Cli 119^1^ | F: GACCTGAAACAGCAATGGG  R: TGTTCTTGAGTCGGTCGG | GA | 180-214 | NED |
| Cac 56^2^ | F: ACCGAGATGCAAAGAGAAGG  R: GTCTTTGGGCAAGCTGTGAG | GA | 221-229 | NED |

| **Table S3** *C. sorrah* null allele frequencies by sampling location and average null allele frequencies of all locations per locus. Grey shading indicates markers that were omitted from our analysis due to null allele frequencies > 5%. | | | | | | |
| --- | --- | --- | --- | --- | --- | --- |
| **Loci** | **Location** | | | | **Average** | **%** |
|  | **Bahrain** | **Oman** | **UAE** | **Yemen** |  |  |
| CS8 | 0.03 | 0 | 0 | 0 | 0.01 | 1 |
| CS40 | 0.05 | 0.09 | 0.07 | 0.04 | 0.06 | 6 |
| CS51 | 0.07 | 0 | 0.06 | 0.03 | 0.04 | 4 |
| CS55 | 0.03 | 0.04 | 0.12 | 0.04 | 0.05 | 5 |
| CS102 | 0 | 0 | 0 | 0 | 0 | 0 |
| CS104 | 0.01 | 0.01 | 0 | 0 | 0.01 | 1 |
| CS295 | 0 | 0.03 | 0 | 0.08 | 0.02 | 2 |
| CS433 | 0 | 0 | 0.03 | 0 | 0.01 | 1 |
| AG11 | 0.06 | 0.03 | 0 | 0 | 0.02 | 2 |
| CS25 | 0 | 0.05 | 0 | 0.02 | 0.01 | 1 |
| CS29 | 0 | 0 | 0 | 0 | 0 | 0 |
| CS94 | 0 | 0.02 | 0 | 0 | 0.01 | 1 |
| CS109 | 0 | 0.02 | 0 | 0 | 0.01 | 1 |
| CS179 | 0.07 | 0.02 | 0.05 | 0.04 | 0.04 | 4 |
| CS288 | 0 | 0 | 0 | 0 | 0 | 0 |
| CS381 | 0.04 | 0.03 | 0 | 0 | 0.01 | 1 |
| CS400 | 0.02 | 0 | 0 | 0 | 0.01 | 1 |

| **Table S4** Mismatch error rates between reference genotypes and re-genotyped replicates. Causes of observed mismatches for *C. sorrah* based on two replicates of 96 samples. Grey shading indicates locus with genotyping error > 5%. | | | | |  |
| --- | --- | --- | --- | --- | --- |
|  | | **Cause of error** | | |  |
| **Loci** | **Mismatch error rate** | **No amplification** | | **Wrong allele call** |  |
| CS8 | 0 | 0 | 0 | |  |
| CS40 | 0.14 | 11 | 3 | |  |
| CS51 | 0.1 | 10 | 0 | |  |
| CS55 | 0.19 | 11 | 8 | |  |
| CS102 | 0.01 | 1 | 0 | |  |
| CS104 | 0.04 | 4 | 0 | |  |
| CS295 | 0.07 | 5 | 2 | |  |
| CS433 | 0.06 | 6 | 0 | |  |
| AG11 | 0.05 | 5 | 0 | |  |
| CS25 | 0.08 | 7 | 0 | |  |
| CS29 | 0.07 | 5 | 1 | |  |
| CS94 | 0.04 | 4 | 0 | |  |
| CS109 | 0.04 | 2 | 2 | |  |
| CS179 | 0.13 | 10 | 2 | |  |
| CS201 | 0.03 | 2 | 0 | |  |
| CS288 | 0.02 | 1 | 0 | |  |
| CS381 | 0.09 | 6 | 2 | |  |
| CS400 | 0.05 | 4 | 0 | |  |
|  |  |  |  | |  |

| **Table S5** Genotyping mismatches among mother-pup genotypes for *C. sorrah.* Grey shading indicates loci with a moderately high rate of genotyping mismatch between mothers (n= 18) and pups (n=78). | | | |
| --- | --- | --- | --- |
| **Marker** | **M-P-mismatch** | **Marker** | **M-P-mismatch** |
| CS 8 | 0 | CS 25 | 0.005 |
| CS40 | **0.02** | CS 29 | 0 |
| CS 51 | 0.005 | CS94 | 0.001 |
| CS 55 | **0.027** | CS 109 | 0 |
| CS 102 | 0 | CS 179 | 0.016 |
| CS104 | 0.005 | CS 201 | 0.005 |
| CS295 | 0.01 | CS 288 | 0 |
| CS 433 | 0.005 | CS 381 | 0.005 |
| AG 11 | 0 | CS 400 | 0.017 |

| **Table S6** *C. limbatus* null allele frequencies estimated for each locus and location. Average and percent null allele frequencies estimated across all locations. (Loci with a > 5% null allele frequency are shaded in grey). B = Bahrain, K = Kuwait, O = Oman, P = Pakistan, SF = South Africa, SR = Saudi Arabia, U = UAE, and Y = Yemen. | | | | | | | | | | |
| --- | --- | --- | --- | --- | --- | --- | --- | --- | --- | --- |
| **Loci** | **Location** | | | | | | | | **Average** | **%** |
|  | **B** | **K** | **O** | **P** | **SF** | **SR** | **U** | **Y** |  |  |
| AC 35 | 0 | 0.2 | 0 | 0 | 0.01 | 0 | 0 | 0 | 0.03 | 3 |
| AC 50 | 0 | 0.03 | 0 | 0 | 0.01 | 0 | 0.02 | 0.05 | 0.01 | 1 |
| AC 60 | 0.1 | 0.09 | 0.09 | 0.3 | 0.05 | 0.07 | 0.09 | 0.2 | 0.12 | **12** |
| AG 2 | 0.1 | 0.05 | 0.1 | 0.2 | 0.1 | 0.01 | 0.01 | 0.1 | 0.08 | **8** |
| AG 5 | 0.04 | 0 | 0.02 | 0 | 0.02 | 0 | 0 | 0 | 0.01 | 1 |
| AG 10 | 0.05 | 0.03 | 0 | 0 | 0.02 | 0 | 0 | 0 | 0.01 | 1 |
| Cli 107 | 0 | 0.04 | 0.02 | 0.02 | 0 | 0.01 | 0.02 | 0 | 0.01 | 1 |
| AC 15 | 0.07 | 0.04 | 0.05 | 0.05 | 0.03 | 0.02 | 0.04 | 0 | 0.03 | 3 |
| AC 17 | 0.1 | 0.2 | 0.05 | 0.1 | 0.05 | 0.04 | 0.06 | 0.06 | 0.08 | **8** |
| AC 27 | 0.09 | 0.08 | 0 | 0.04 | 0.01 | 0.03 | 0.04 | 0.03 | 0.04 | 4 |
| AC 47 | 0 | 0.09 | 0.01 | 0 | 0 | 0 | 0 | 0 | 0.01 | 1 |
| AG 11 | 0.03 | 0 | 0 | 0.04 | 0 | 0 | 0 | 0 | 0.01 | 1 |
| Cac 56 | 0 | 0 | 0 | 0 | 0 | 0 | 0 | 0 | 0 | 0 |
| Cli 119 | 0.03 | 0 | 0 | 0 | 0 | 0 | 0 | 0 | 0.003 | 0.3 |

| **Table S7** Mismatch error rates between reference genotypes and re-genotyped replicates for *C. limbatus*. Causes of observed mismatches for based on two replicates of 96 samples. Grey shading indicates loci with genotyping error > 5%. | | | |
| --- | --- | --- | --- |
|  |  | **Cause of error** | |
| **Loci** | **Mismatch error rate** | **No amplification** | **Wrong allele call** |
| **AC 35** | 0 | 0 | 0 |
| **AC 50** | 0.04 | 3 | 0 |
| **AC 60** | 0.16 | 12 | 3 |
| **AG 2** | 0.07 | 5 | 1 |
| **AG 5** | 0 | 0 | 0 |
| **AG 10** | 0.04 | 3 | 0 |
| **Cli 107** | 0.02 | 1 | 0 |
| **AC 15** | 0.05 | 3 | 1 |
| **AC 17** | 0.09 | 3 | **5** |
| **AC 27** | 0.06 | 4 | 1 |
| **AC 47** | 0.01 | 1 | 0 |
| **AG 11** | 0 | 0 | 0 |
| **Cac 56** | 0 | 0 | 0 |
| **Cli 119** | 0 | 0 | 0 |

| **Table S8** *P*-values of *C. sorrah* heterozygosity excess under the three models tested in BOTTLENECK; IAM = Infinite Allele Model, TPM = Two-Phase Model, SMM = Single-Step Mutation Model. | | | | | |
| --- | --- | --- | --- | --- | --- |
|  | **Bahrain** | **UAE** | **Oman** | | **Yemen** |
| **H_E_ excess** |  |  | |  |  |
| **IAM** | 0.06 | 0.002 | | 0.07 | 0.008 |
|  |  |  | |  |  |
| **TPM** | 0 | 0 | | 0 | 0.01 |
|  |  |  | |  |  |
| **SMM** | 0 | 0 | | 0 | 0 |

| **Table S9** *P*-values of *C. limbatus* heterozygosity excess under the three models tested in BOTTLENECK; IAM = Infinite Allele Model, TPM = Two-Phase Model, SMM = Single-Step Mutation Model. | | | | | | | | |
| --- | --- | --- | --- | --- | --- | --- | --- | --- |
|  | **Bahrain** | **Kuwait** | **Oman** | **Pakistan** | **SAF** | **SAR** | **UAE** | **Yemen** |
| **H_E_ excess** |  |  |  |  |  |  |  |  |
| **IAM** | 0.6 | 0.5 | 0.6 | 0.3 | 0.05 | 0.05 | 0.5 | 0.4 |
|  |  |  |  |  |  |  |  |  |
| **TPM** | 0.01 | 0.08 | 0.01 | 0.08 | 0.004 | 0.02 | 0.003 | 0.003 |
|  |  |  |  |  |  |  |  |  |
| **SMM** | 0.01 | 0.02 | 0.002 | 0.02 | 0 | 0.001 | 0.002 | 0.001 |

| **Table S10** Average monthly and yearly mean sea surface temperatures of locations sampled in The Gulf, the Sea of Oman and the Gulf of Aden. Grey and blue shading indicate monthly minimum and maximum temperatures, respectively (World Sea Temperature, 2015). *Variance (s^2^)* = variance of the average annual change in sea surface temperatures. | | | | | |
| --- | --- | --- | --- | --- | --- |
|  | **Bahrain** | **Kuwait** | **Oman** | **UAE** | **Yemen** |
| January | 19 | 16 | 24 | 22 | 25 |
| February | 18 | 15 | 23 | 20 | 25 |
| March | 20 | 18 | 24 | 22 | 26 |
| April | 22 | 21 | 26 | 25 | 28 |
| May | 27 | 26 | 30 | 29 | 30 |
| June | 30 | 29 | 31 | 31 | 30 |
| July | 32 | 31 | 31 | 32 | 28 |
| August | 33 | 32 | 30 | 33 | 28 |
| September | 32 | 31 | 30 | 33 | 29 |
| October | 30 | 26 | 29 | 31 | 28 |
| November | 26 | 24 | 27 | 28 | 27 |
| December | 22 | 19 | 25 | 24 | 26 |
| **Mean ± S.E.** | 25.9 ± 1.7 | 24 ± 1.8 | 27.5 ± 0.9 | 27.5 ± 1.4 | 27.5 ± 0.5 |
| ***Variance (s^2^)*** | 30 | 37 | 9 | 22 | 3 |

**Figure S1** STRUCTURE analysis results showing Ln P(D) and ΔKvalues based on five replicates for each value of K (K 1-10). A) *C. sorrah*, and B) *C. limbatus.*

**Figure S2 Neighbour joining tree based on the** *F*_ST_ genetic distance matrix for *C. sorrah*. Values on tree branches are pairwise *F*_ST_ values.

**Figure S3 Neighbour joining tree based on the** *F*_ST_ genetic distance tree for *C. limbatus.* Values on tree branches are pairwise *F*_ST_ values.

**Figure S4** Difference in the values of the corrected Assignment Index (*AI_c_*) between males and females for *C. sorrah* (A) *C. limbatus* (B).

**References:**

Giresi, M., Renshaw, M. A., Portnoy, D. S. and Gold, J. R. (2012). Isolation and characterization of microsatellite markers for the blacknose shark, *Carcharhinus acronotus*. *Conservation Genetics Resources*, *4*(1), 141-145.

Keeney, D. B. and Heist, E. J. (2003). Characterization of microsatellite loci isolated from the blacktip shark and their utility in requiem and hammerhead sharks. *Molecular Ecology Resources*, *3*(4), 501-504.

World Sea Temperature. Available at: https://www.seatemperature.org/ (accessed March 20 2018).
